# Gene expression patterns through oral squamous cell carcinoma development: PD-L1 expression in primary tumor and circulating tumor cells

**DOI:** 10.18632/oncotarget.3939

**Published:** 2015-05-15

**Authors:** Joao Paulo Oliveira-Costa, Alex Fiorini de Carvalho, Giorgia Gobbi da Silveira, Peter Amaya, Yongqi Wu, Kyoung-Joo Jenny Park, Mabel Pinilla Gigliola, Maryam Lustberg, Marcilei Eliza Cavicchioli Buim, Elisa Napolitano Ferreira, Luiz Paulo Kowalski, Jeffrey J. Chalmers, Fernando Augusto Soares, Dirce Maria Carraro, Alfredo Ribeiro-Silva

**Affiliations:** ^1^ Department of Pathology, Ribeirao Preto School of Medicine, University of Sao Paulo, Ribeirao Preto, Brazil; ^2^ Laboratory of Genomics and Molecular Biology, CIPE, A.C. Camargo Cancer Center, Sao Paulo, Brazil; ^3^ William G. Lowrie Department of Chemical and Biomolecular Engineering, The Ohio State University, Columbus, OH, USA; ^4^ Stefanie Spielman Comprehensive Breast Center, Wexner Medical Center, The Ohio State University, Columbus, OH, USA; ^5^ Department of Anatomic Pathology, A.C. Camargo Cancer Center, Sao Paulo, Brazil; ^6^ Department of Head and Neck Surgery and Otorhinolaryngology, A.C. Camargo Cancer Center, Sao Paulo, Brazil

**Keywords:** PD-L1, oral squamous cell carcinoma, gene expression, circulating tumor cells, survival

## Abstract

Oral squamous cell carcinoma (OSCC) is the most common tumor of the oral cavity and has been associated with poor prognosis. Scarce prognostic markers are available for guiding treatment and/or sub-classifying patients. This study aims to identify biomarkers by searching for genes whose expression is increased or decreased during tumor progression (through T1 to T4 stages). Thirty-six samples from all tumor size stages (from T1 to T4) were analyzed using cDNA microarrays. Selected targets were analyzed by immunohistochemistry and in circulating tumor cells by immunofluorescence and Nanostring. Correlation was shown between PD-L1 and tumor size and lymph node metastasis, HOXB9 and tumor size, BLNK and perineural invasion, and between ZNF813 and perineural invasion. PD-L1 positivity was an independent prognostic factor in this cohort (*p* = 0.044, HH = 0.426). In CTCs from patients with locally advanced OSCC, we found a strong cytoplasmatic expression of PD-L1. PD-L1 is a ligand of PD-1 and is believed to limit T cell activity in inflammatory responses and limit autoimmune diseases. We demonstrated an important role for PD-L1 in primary tumors according to tumor size, and in disease specific survival. Therefore, we could further determine individuals with PD-L1+ CTCs, and possibly follow treatment using CTCs.

## INTRODUCTION

Oral squamous cell carcinoma (OSCC) is the most common type of malignant tumor in the head and neck area and has been associated with poor prognosis and a higher morbidity when diagnosed at advanced stages [1]. One of the most important risk factors is the abusive use of both tobacco and alcohol, which leads to a 11-fold increase in the development of the disease [2]. OSCC is believed to originate from sequential mutations that can develop as a consequence of progressive genetic instability acquired over time. The identification of reliable biomarkers that can determine a better or worst prognosis is a continuous challenge. Overall mortality rate of OSCC patients has remained unchanged at approximately 50% over the last several decades, even considering the recent advances in therapies and research [3–6]. Due to the relative unreliability of current prognostic factors, such as TNM staging, there is considerable interest in discovering prognostic biomarkers that can help to discriminate patients and guide effective therapeutic strategies [3, 4, 6].

Nowadays, treatment is generally based on surgical resection of primary tumor, which may be followed by radio- and chemotherapy [7]. The selection of post-surgical treatment relies in factors such as histological grading, tumor location, radiographic findings, and the TNM system grading. The TNM system grading was proposed by Union for International Cancer Control back in 1968 and takes into consideration the tumor size (T), lymph node status (N), and the presence of distant metastasis (M) [8]. Staging systems mainly has four basic functions: to estimate patients’ survival odds, to help to better determine treatment, to evaluate treatment efficacy, to define a nomenclature pattern, and to allow the comparison of results between different institutions [9]. While it has been important in cancer treatment planning and reporting results through the decades, the TNM staging system still has some flaws, such as patients with the same TNM stage having different clinical behaviors, different treatment responses, and even different outcomes. Several biological factors could help either substitute or complement TNM cancer classification in patients.

Several studies have tried to better classify OSCC, similar to what has been achieved in breast tumors, where at least five different molecular patterns have been recognized since cDNA microarrays have been used to provide a molecular portrait of these tumors [10]. Although in breast cancer, these molecular profiles were shown to be important either as prognostic indicators or important to determine different treatment schemes, in OSCC (and in squamous tumors in general) the molecular classification of tumors has not been successful, and it is impossible to molecularly classify OSCC despite some efforts that have been made. Chung and collaborators have shown that OSCC could be divided in four different groups, being one of them similar to basal breast tumors, another showing a mesenchymal signature, a third showing positivity to cytokeratin 14, and the last one resembling a molecular signature similar to that seen in tobacco-exposed patients [11]. But different from breast tumors, this classification was not further tested or widely implemented in the clinical practice.

Therefore, the aim of this study is to verify differences in expression between tumors of different T stages, looking for genes with either an increase or decrease through T1 to T4 stages, and to further carefully evaluate the transcriptional and protein levels of these genes in independent cohorts for identifying potential biomarkers.

## RESULTS

### Patients and samples

A total of 36 OSCC frozen samples were retrieved from AC Camargo Cancer Center Biobank. The median size of the tumor was 3.214 cm (standard mean error 0.2512). The ratio between male and female was 3.66:1 and the mean age was 61.80 years (ranging from 34 to 95 years). Sixteen out of 36 samples showed perineural invasion in the specimen, while two showed vascular invasion and 6 presented with lymphatic invasive disease. Lymph node metastases were confirmed in 17 patients, with a mean of 2.62 positive lymph nodes per patient.

### Microarray data filtering and profiling

**Figure 1:**
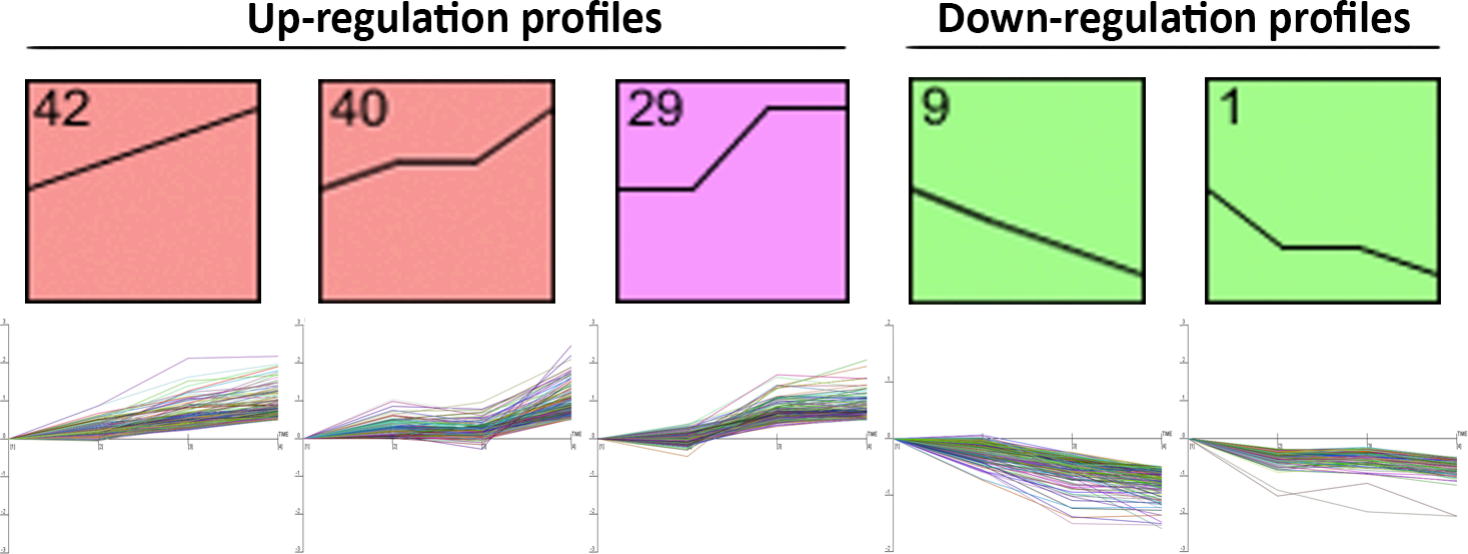
Panel representing the Short Time-series Expression Miner (STEM) analysis profiles, which were used to cluster and analyze the expression data

To determine expression profiles of different-sized tumor, we have analyzed whole genome expression through microarray platform containing coding and long intergenic non-coding RNAs (lincRNAs). After filtering emission intensity data in GeneSpring 12.6 software (Agilent Technologies), each case was classified according to its T stage, and all filtered genes were used for hierarchical clustering analysis based on genes with different gene expression between T1 and T4 stages. After this first analysis, we processed the remaining genes in STEM. A total of 3503 coding RNAs and 56 lincRNAs were included in STEM analysis. After STEM analysis, we chose 5 different profiles with 879 transcripts whose expression was significantly increased or decreased from T1 to T4 stage. Profile 42 (186 genes assigned, 74.8 genes expected; *p*-value = 4.3^−28^), profile 40 (192 genes assigned, 89.2 genes expected; *p*-value = 6.3^−22^), profile 9 (176 genes assigned, 74.7 genes expected; *p*-value = 4.0^−24^), profile 1 (139 genes assigned, 60.7 genes expected; *p*-value = 2.4^−18^) and profile 29 (186 genes assigned, 99.9 genes expected; *p*-value = 3.5ˆ^−15^) were selected from STEM, and selected genes between these profiles were further analyzed (Figure 1).

### RT-qPCR and clinicopathological data

For technical validation, from the 879 transcripts whose expression was modulated during tumor progression, we selected six for assessing in RT-qPCR in the same cohort used in microarray analysis. Due to the large number of genes selected from filtering and profiling analysis, we chose to focus on genes showing either increasing or decreasing profiles according to tumor size.

PD-L1, HOXB9 and DHDH were selected from profiles 42, 40 and 29, while BLNK, ZNF813 and IL6ST were selected from profiles 9 and 1. Classifying the different transcripts as down-regulated, non-modulated or up-regulated, we found a significant correlation between PD-L1 and tumor size (*p* = 0.029), and lymph node status (*p* = 0.026). We also found a correlation between HOXB9 expression and tumor size (*p* = 0.021) and gender (*p* = 0.006), BLNK and perineural invasion (*p* = 0.023) and ZNF813 and perineural invasion (*p* = 0.029). All significant associations are shown in Table 1.

**Table 1:** Relationship between PD-L1, HOXB9, BLNK and ZNF813 nas clinical and pathological parameters, and their distribution according to variables

	**Tumor Size**		**<4 cm**	**>4 cm**	**Total**	***p*-value**
	Down-regulated		13	5	18	0.029
PD-L1	Normal		4	4	8	
	Up-regulated		3	7	10	
Total			20	16	36	
	**Lymph Node Metastasis**		**Yes**	**No**	**Total**	***p*-value**
	Down-regulated		12	4	16	0.026
PD-L1	Normal		2	6	8	
	Up-regulated		3	6	9	
Total			17	16	33	
	**Tumor Size**		**<4 cm**	**>4 cm**	**Total**	***p*-value**
HOXB9	Normal		5	0	5	0.021
	Up-regulated		11	14	25	
Total			16	14	30	
	**Gender**		**Male**	**Female**	**Total**	***p*-value**
HOXB9	Normal		1	2	3	0.006
	Up-regulated		22	2	24	
Total			23	4	27	
	**Perineural Invasion**		**Yes**	**No**	**Total**	***p*-value**
	Down-regulated		10	5	15	0.023
BLNK	Normal		5	9	14	
	Up-regulated		1	5	6	
Total			16	19	35	
	**Perineural Invasion**		**Yes**	**No**	**Total**	***p*-value**
	Down-regulated		14	11	25	0.029
ZNF813	Normal		2	4	6	
	Up-regulated		0	4	4	
Total			16	19	35	

### Interaction networks

**Figure 2:**
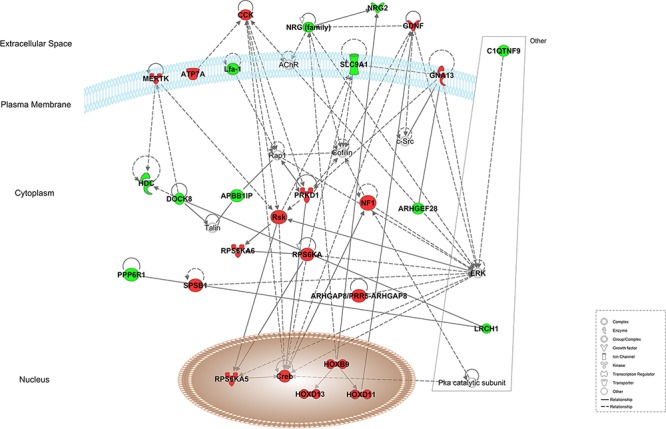
Representation of network 4 (which includes HOXB9) Up- and down-regulated genes in red and green, respectively.

**Figure 3:**
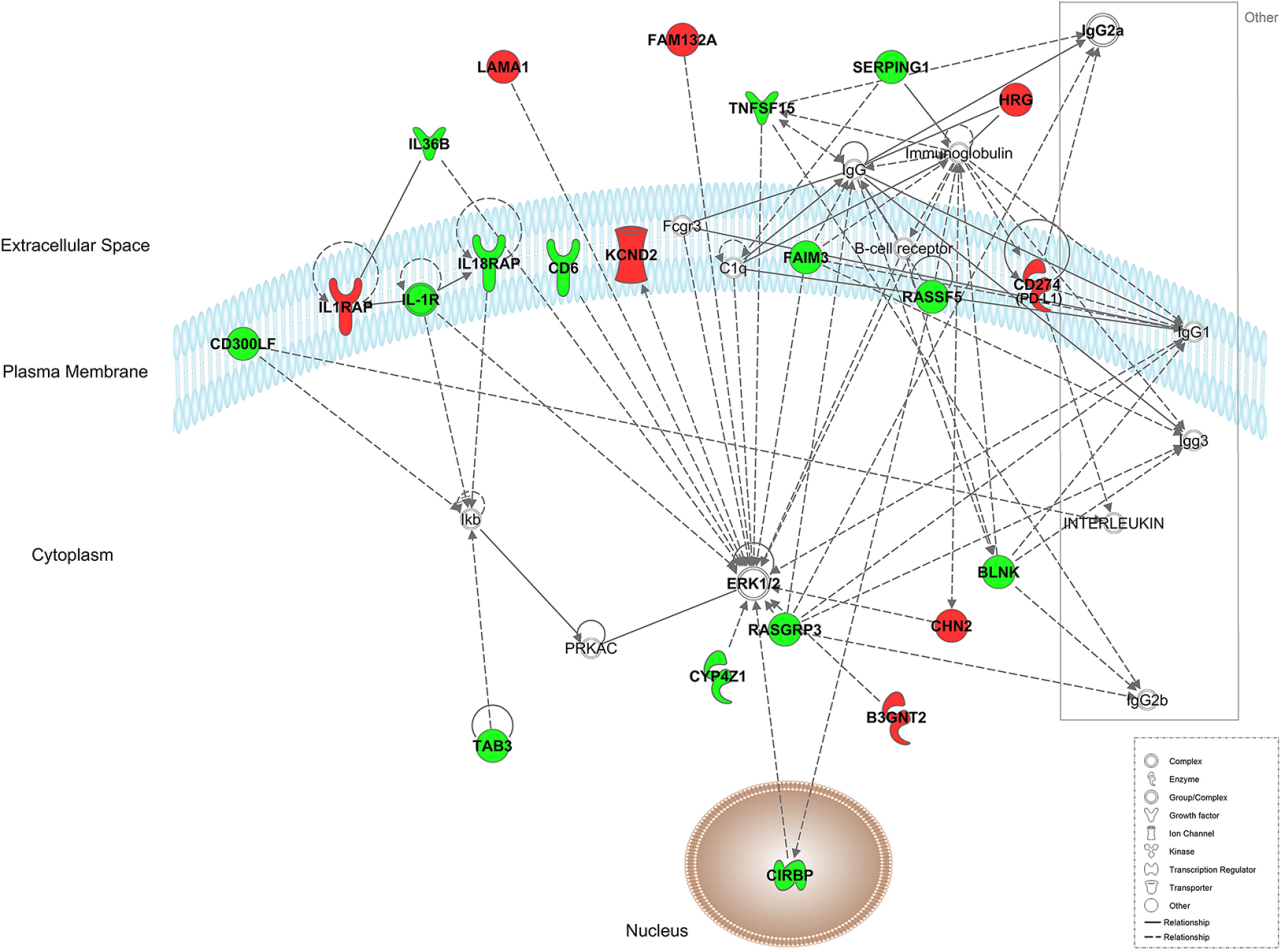
Representation of network 5 (which includes PD-L1 (CD274) and BLNK) Up- and down-regulated genes in red and green, respectively.

**Figure 4:**
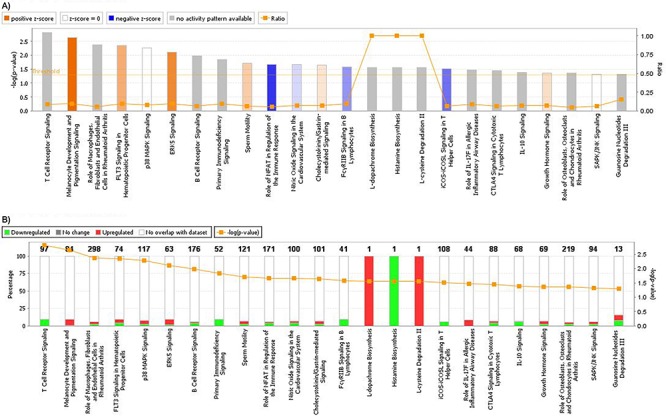
A. Graphic representation of canonical pathways according to OSCC differentially expressed genes In y axis, bars represent −log (*p*-value) (the larger the value, lesser is *p*-value). Bar colors represent z-score, where orange bars represent a significant increase in a given biological function; blue bars represent a significant decrease in biological function and grey bars show no significant increase or decrease. Orange lines and right y axis represent ratio, that is the ratio between the number of genes of your dataset relative to the total number of genes in a given pathway. **B.** Graphic representation of genes included in each pathway. Green shows downregulated genes, while red represents upregulated genes, and y axis shows the percentage of down regulated and unregulated genes, compared to the total number of genes in a given pathway. Right y axis and orange line shows −log (*p*-value).

**Figure 5:**
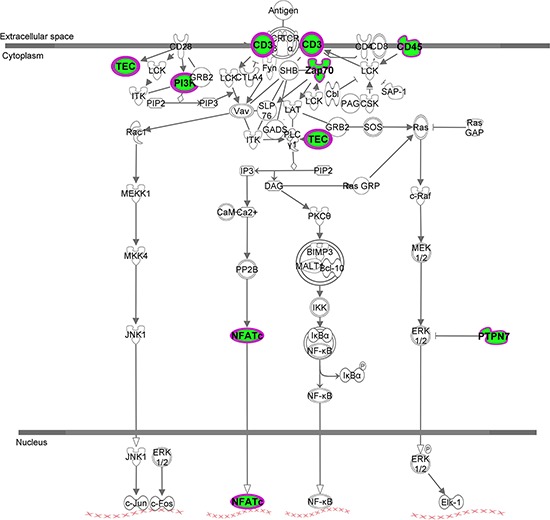
T cell receptor signaling canonical pathway obtained by IPA Up- and down-regulated genes in red and green, respectively

Using Ingenuity Pathway Analysis software (Qiagen, Venlo, The Nederlands), a list of downregulated and upregulated genes was analyzed, and 25 interaction networks were found to be involved, according to the genes included (Supplementary Table 1). Based on the relationships found in RT-qPCR analysis we chose the interaction networks involving PD-L1, BLNK and HOXB9 (Figures 2, 3). The most well represented canonical pathway within our gene list was “T cell receptor pathway” (*p*-value = 0.002) (Figures 4, 5 and Supplementary Table 2).

### Immunohistochemical analysis and clinicopathological data

A total of 142 specimens were used for our biological validation, using the inclusion criteria and were therefore selected for immunohistochemical (IHC) analysis. From the initial 142 cases, some had missing information, such as size, or tobacco and alcohol consumption, and others had no material in their TMA slide, and were excluded from further analysis. Statistical analyses were based on the number of cases with all the information in each separate cross table. The mean age of the patients in our cohort was 57.89 years, with ages ranging from 32 to 90 years, with a male to female ratio of 7.35:1. A significant percentage of the OSCC patients had reported simultaneous tobacco and alcohol consumption (96 of 131; 73.2%), although for 11 cases, there was no information about smoking and alcohol intake. Regarding histological grading, 62 patients (43.7%) showed well-differentiated tumors, 65 patients (45.8%) showed moderately differentiated tumors, and 13 patients presented poorly differentiated tumors. All demographic and clinical data of this cohort is summarized in Table 2.

**Table 2:** Relationship between PD-L1, HOXB9 and clinico-pathological parameters and their distribution according to variables

		Cytoplasm			Membrane			Nuclear		
		PD-L1 +	PD-L1 −	*p*-value	PD-L1 +	PD-L1 −	*p*-value	HOXB9 +	HOXB9 −	*p*-value
Local	Tongue	11	16	0.678	1	26	n/c	27	4	0.775
	Floor of the mouth	14	11		0	25		23	1	
	Inferior lip	6	6		1	11		10	0	
	Palate	3	6		1	8		10	3	
	Bucal Mucosa	4	2		1	5		6	0	
	Retromolar trigone	8	5		2	11		11	2	
	Gingiva	1	3		1	3		4	0	
Gender	Male	42	43	0.807	7	0	0.328	80	10	0.248
	Female	5	6		78	11		11	0	
Age	<60	32	30	0.313	3	59	0.216	62	4	0.770
	>60	15	19		4	30		29	6	
Tumor Size	<4	29	29	0.382	3	55	0.800	54	6	0.192
	>4	10	6		3	13		16	0	
Smoking	Yes	38	40	0.139	7	71	0.306	74	9	0.794
	No	8	3		0	11		11	1	
Alcohol	Yes	36	33	0.714	7	62	0.151	69	4	0.002
	No	9	10		0	19		15	6	
Lymph node Metastasis	Yes	19	24	0.354	4	39	0.517	39	4	0.950
	No	28	24		3	49		51	5	
Distant Metastasis	Yes	3	3	0.979	1	5	0.373	6	0	0.429
	No	44	45		6	86		84	9	
Histological Grading	I	25	21	0.204	6	40	0.50	39	6	0.429
	II	18	22		1	39		41	3	
	III	3	6		0	9		10	1	

### PD-L1 protein and clinicopathological data

**Figure 6:**
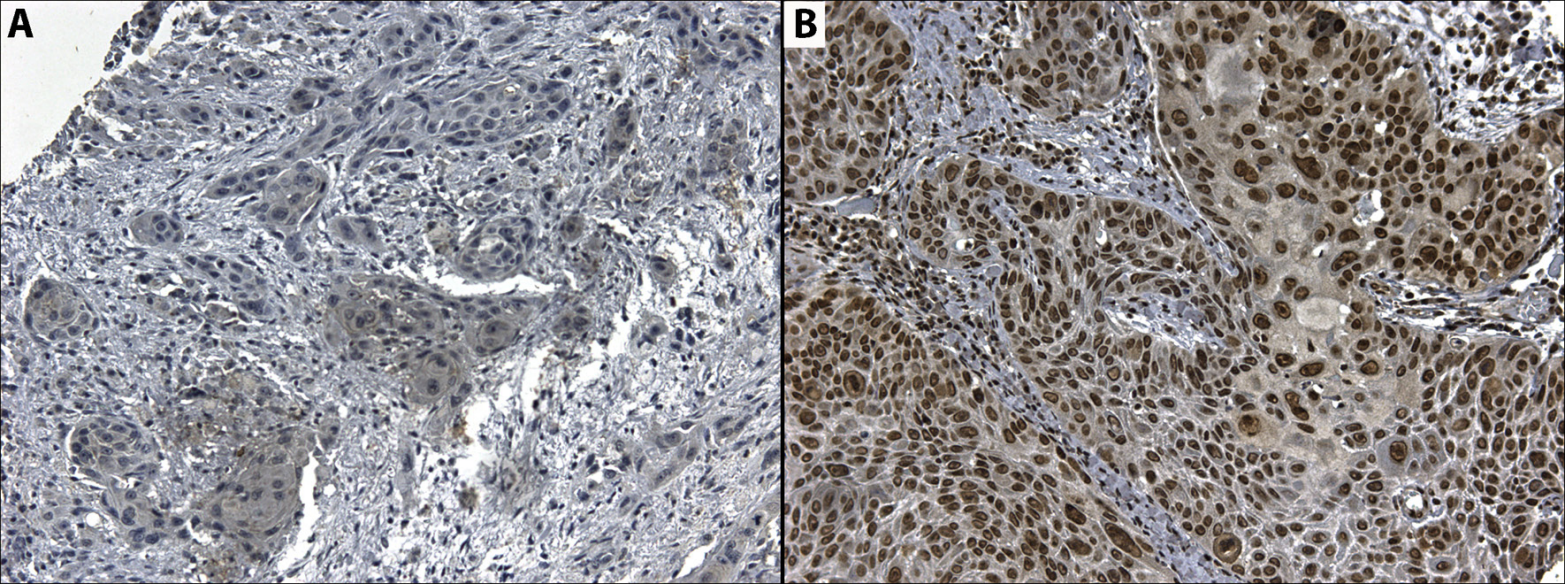
Microphotography of A. Cytoplasmic expression of CD274 in a positive case, and B. Nuclear expression of HOXB9 in a positive case Magnification 20x.

PD-L1 expression was observed either in the cytoplasm or the plasma membrane of OSCC cells, in agreement with the findings of other authors and catalogued in Human Protein Atlas (http://www.proteinatlas.org). The cytoplasmic expression of PD-L1 was observed in 47 cases out of 96 (49%) while membrane expression was observed in 7 cases out of 96 (7.3%). PD-L1 cytoplasmic expression was histologically found in areas with undifferentiated cells, especially those with atypical nuclei and in keratin. HOXB9 nuclear expression was found in 91 out of 96 cases (90.1%). The relationship between PD-L1 and HOXB9 expression and clinicopathological data are shown in Table 2. PD-L1 and HOXB9 expression are shown in Figure 6. No association was found between either PD-L1 or HOXB9 expression and clinicopathological parameters.

### Survival analysis

**Figure 7:**
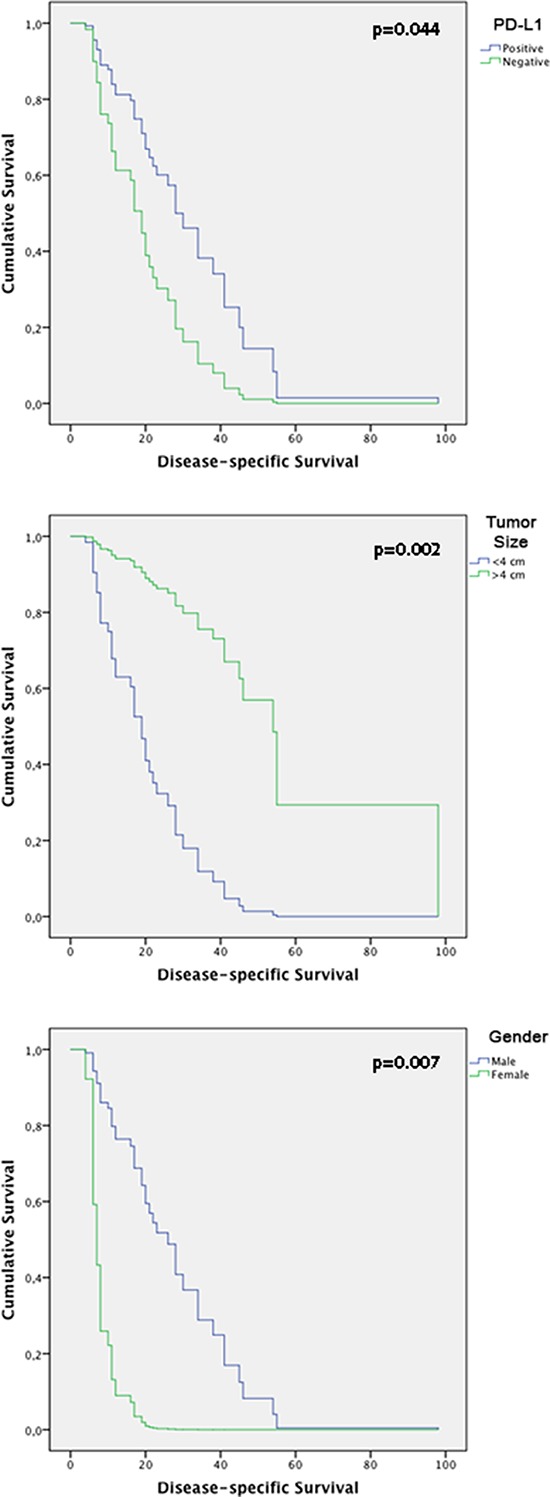
Kaplan-Meier tables obtained from multivariate analysis according to time until death from HNSCC, showing CD274, gender and tumor size effect on disease-specific survival

The follow-up period for the OSCC patients was used to determine survival rates and ranged from 4 to 108 months (mean, 20 months). The median survival time was 15.5 months. The 5-year disease specific survival rate was 40%. Cox's Proportional Hazards multivariate analysis identified cytoplasmic PD-L1 [*p* = 0.044; HR: 0, 426 (C.I.: 0.186–0.977)], tumor size [*p* = 0.002; HR: 7.618 (C.I.: 2.139–27.128)] and gender [*p* = 0.007; HR: 0.112 [(C.I.: 0.022–0.554)] as independent prognostic markers shown in Table 3. Kaplan-Meier tables are presented in Figure 7.

**Table 3:** Cox proportional hazards model according to time until death due to OSCC

Variables in the Equation				
	*p*-value	HR	95.0% IC for HR	
			Lower	Upper
Tongue	0,629	-	-	-
FOM	0,546	1,669	0,316	8,816
Inferior lip	0,292	2,570	0,443	14,895
Palate	0,390	2,129	0,380	11,927
Buccal Mucosa	0,177	5,866	0,449	76,699
Retromolar trigone	0,366	3,708	0,216	63,522
Gingiva	0,817	0,809	0,134	4,865
Cytoplasmic PD-L1	**0,044**	0,426	0,186	0,977
Membranous PD-L1	0,270	2,628	0,473	14,613
HOXB9	0,189	6,699	0,393	114,285
Gender	**0,007**	0,112	0,022	0,554
Age	0,477	0,691	0,250	1,914
Smoking	0,632	1,621	0,224	11,731
Alcohol	0,996	1,005	0,159	6,361
Size	**0,002**	7,618	2,139	27,128
Lymph Node metastasis	0,320	0,593	0,211	1,662
Distant metastasis	0,652	0,525	0,032	8,654
Histologic Grading - Well differentiated	0,381	-	-	-
Histologic Grading - Moderately differentiated	0,439	2,668	0,222	32,081
Histologic Grading - Poorly differentiated	0,740	1,581	0,105	23,702

F.O.M: Floor of mouth

### Nanostring analysis in CTCs

**Figure 8:**
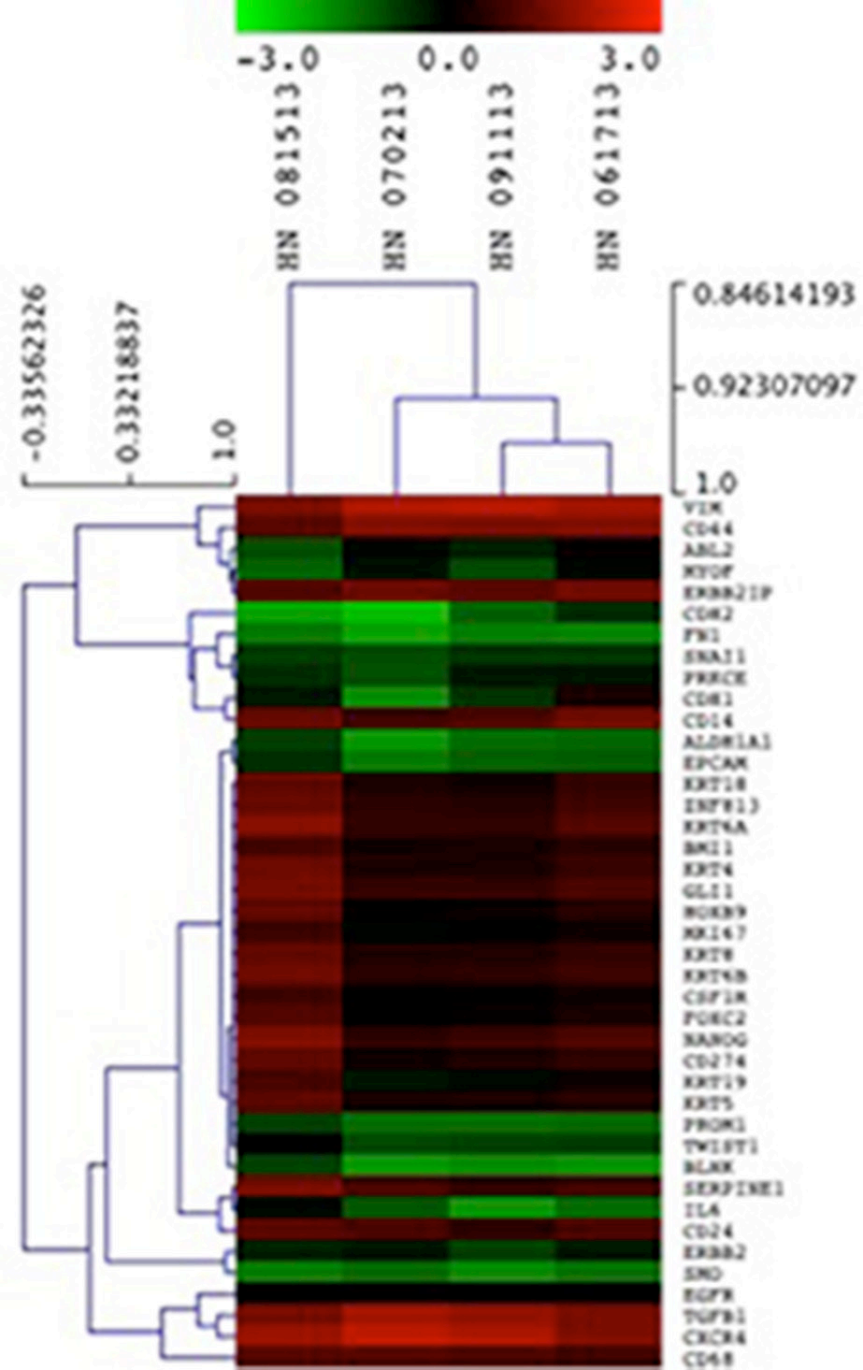
Unsupervised hierarchical clustering of CTCs isolated from HNSCC samples using epithelial markers, CD274, HOXB9, BLNK and ZNF813

Following the results of the RT-PCR analysis, we sought to investigate PD-L1, HOXB9, BLNK and ZNF813 in CTCs from patients from The Ohio State University Wexner Medical Center. Nanostring analysis was performed on CD45 negative enriched cells. All four samples used in Nanostring analysis showed expression of the four markers tested. Comparing the CTC samples with healthy blood donors, we found a mean fold change of −1, 4 for BLNK, 0.6 for PD-L1, 0.45 for HOXB9 and 0.74 for ZNF813. It was interesting to note that the expression pattern was similar to that found in primary tumors in our first cohort, with a discrete increase in PD-L1, HOXB9 and ZNF813 and an important decrease in BLNK expression. The heat map and expression data are shown in Table 4. Clustered genes according to samples are shown in Figure 8.

**Table 4:** Heat map showing gene expression of target genes PD-L1, HOXB9, ZNF813 and BLNK, besides citokeratins, EPCAM and Vimentin in circulating tumor cells from OSCC patients

Gene Symbol	HN 081513	HN 070213	HN 061713	HN 091113
BLNK	−0,8	−1,8	−1,7	−1,6
PD-L1	1,2	0,2	0,6	0,4
EPCAM	−0,6	−1,5	−1,1	−1,2
HOXB9	1,2	0,1	0,5	0,1
KRT18	1,2	0,3	0,6	0,2
KRT19	0,9	−0,3	0,2	−0,1
KRT4	1,3	0,3	0,7	0,3
KRT5	1,1	0,3	0,5	0,3
KRT6A	1,6	0,6	1	0,6
KRT6B	1,4	0,5	0,7	0,5
KRT8	1,2	0,4	0,6	0,4
VIM	1,5	2,1	1,9	2,2
ZNF813	1,2	0,5	0,7	0,4

### Morphological analysis of PD-L1 protein expression in CTCs

**Figure 9:**
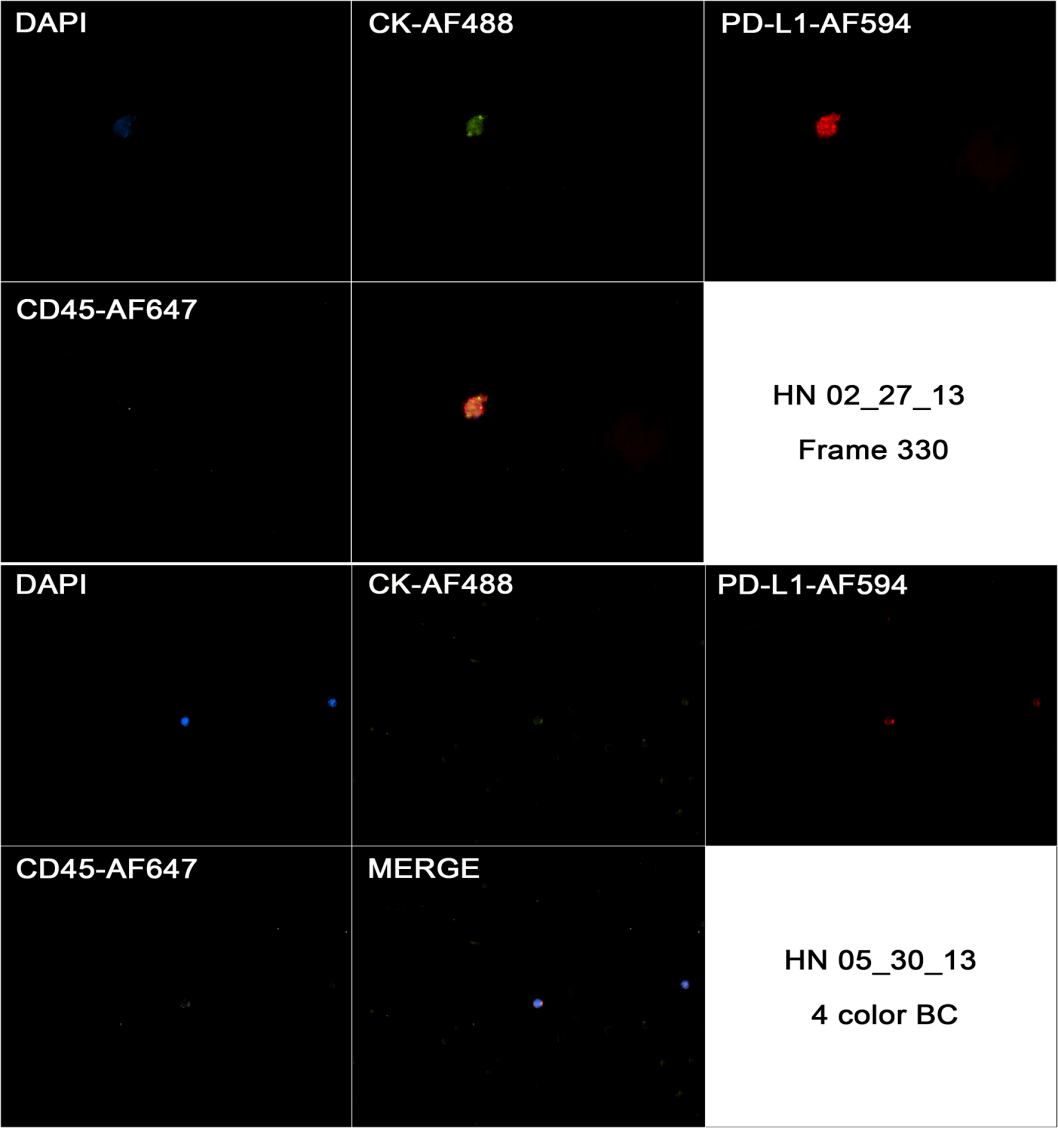
Captured images from a CTC in a HNSCC case (HN 02/27/13) showing positivity to CK, negativity for CD45 and strong and focal positivity to CD274 in first image (frame 330), and the same markers in a blood cell from a different case (HN 05/30/13)

Multiplexed fluorescence analysis was performed in four different samples from patients from The Ohio State University Wexner Medical Center and in all of them we found CD45-CK+ cells with PD-L1 expression. While CD45-CK+ cells showed a strong and focal PD-L1 expression (also cytoplasmic background expression similar to the pattern found in primary tumor). PD-L1 expression was expected in blood cells due to its role as a T cell regulator, but it was interesting to see that PD-L1 had a stronger staining pattern in CTCs when compared to blood cells. PD-L1 expression in CTCs and blood cells are exemplified in Figure 9.

## DISCUSSION

To the best of our knowledge, this is the first study to show different profiles of genes in OSCC tumors with different sizes, ranging from stage T1 to T4, and also to show that significantly expressed genes could be prognostic factors in a different cohort, and also measured in CTCs. PD-L1, shown to be a prognostic factor in our cohort, is also an important target for drugs currently in the pharma pipeline, specifically in Phase I and II clinical trials, and is now shown to play an important role in OSCC development, being expressed in CTCs from locally advanced OSCC patients.

Here we show that three major clusters represent genes with a similar behavior, with either an increase or decrease according to tumor T stage. This finding is important if we take into consideration that “shotgun” approaches, such as cDNA microarrays, sequencing, and other whole transcriptome analyses are not widely used in OSCC research. Thus, only a few research groups were able to perform a whole genome analysis in OSCC samples. One of the most important features of whole genome analysis is the ability to provide further insights into previously unrelated pathways, not studied in a given tumor before. A good example is our finding linking PD-L1 expression with OSCC development.

CD274, or PD-L1 (Programmed Death Ligand-1), is a ligand of the PD-1 molecule, whose expression was first described in murine T cells, B cells, dendritic cells and mesenchymal stem cells [12, 13]. PD-L1 limits T cell activity in peripheral tissues in inflammatory responses and to limit autoimmune diseases [14]. Chronic exposure to antigens can also cause a rise in PD-1 levels, apparently inducing an exhaustion state in T cells, which can be reversed by blocking PD-L1 [15]. This exhaustion state is particularly important in diseases that are impacted by a low CD8+ T cell response, leading to a faster progression of the disease. Previous studies show that CD8+ T cells from HIV-positive individuals have an over-expression of PD-L1, and that this over-expression is related to an exhaustion of T cells and disease progression [16].

Although several types of tumors exhibit PD-L1 over-expression, and this over-expression is related with adverse prognosis and worse overall survival, our findings show that in OSCC, the low expression of PD-L1 is related with worse prognosis as an independent prognostic factor. This result is worrisome when it is taken into consideration that PD-L1 has been used as a target for vaccines and antibodies, and the results are promising from patients over-expressing PD-L1. In 296 patients with previously treated tumors (melanoma, renal cell carcinoma, non-small cell carcinoma of the lung, castration-resistant prostate cancer and colon cancer), an antibody against PD-1 was applied every two weeks, in 12 cycles. Encouraging responses were found in melanoma (28%), renal cell (27%) and pulmonary tumors (18%). Also, the type of response seemed to be long lasting, since 20 patients showed a response lasting for more than 12 months, which was the last informed follow-up [17]. This means that the response could last even more than a year. Unfortunately, no OSCC patients were enrolled, which does not allow us to make any comparisons between our results. The same group has recently reported the results from a different trial. This time, the treatment scheme was performed with escalated doses between 0.3 and 10 mg/kg of an anti-PD-L1 antibody for 207 patients with advanced tumors (melanoma, renal cell carcinoma, non-small cell carcinoma of the lung, ovarian cancer, pancreatic cancer, gastric cancer and breast tumors). The most impressive result came from non-small cell carcinoma of the lung, with a known resistance to immunotherapy. Five out of 49 patients showed objective response, with a 23% rate of disease stabilization in 6 months, in squamous cell subtype [18].

Only a few studies have sought to investigate PD-L1 expression in OSCC, and a high percentage of tumors are classified as positive although these studies does not evaluate transcriptional expression levels [19, 20]. Our results show a positivity rate around 49% for cytoplasmic PD-L1 in OSCC. Also, in multivariate analysis, PD-L1 was shown to be an independent prognostic factor in a different cohort of OSCC patients. In normal conditions, higher PD-L1 levels were expected to help the tumor cells to overcome immune surveillance, due to the expected exhaustion state of T cells. Nevertheless, in carcinogenesis, it seems like PD-L1 over-expression is also related to a better prognosis in non-small cell lung carcinomas. Velcheti and co-workers recently reported a survival prediction for mRNA and protein from PD-L1 in two different cohorts, in a total of 544 cases [21]. It is important to emphasize that the authors used only membranous staining for PD-L1 status, which could happen due to the specificities from the antibody used in their study. As stated in Methods, we have used polyclonal antibody for PD-L1, as a previous study in colorectal carcinomas have shown that either monoclonal or polyclonal antibodies were able to predict colorectal cancer patients overall survival in the same manner [22].

Our observations are consistent with two recent studies in metastatic melanomas and Merkel-cell carcinomas showing association of PD-L1 protein expression with increased tumor infiltrating lymphocytes and survival [23, 24]. Moreover, a recent study in a large colorectal cancer cohort found positive association between PD-L1 protein positivity, tumor infiltration by CD8+ T cells and overall survival [22]. Although the effects of PD-L1 signaling in the tumor are not completely understood, they are likely to be affected by the presence of specific immune regulatory cells and co-activation of additional immune checkpoint pathways. Hamanishi and collaborators demonstrated that tumor infiltration by CD8+ T lymphocytes was associated with increased FOXP3, regulatory T cells and PD-L1 protein and mRNA in metastatic melanomas [25]. In the same work, induction of these co-inhibitory pathways in the tumor microenvironment required the presence of CD8+ T cells and interferon gamma expression in a murine melanoma model [25]. The finding that “T cell receptor pathway” is the most representative canonical pathway regarding all of the differentially expressed genes is an important indication that T cell activation might be of importance in OSCC pathogenesis during T stage changes. T cell receptor signaling is important in developing thymocytes for the establishment of CD4 and CD8 lineages, which are essential for the interaction with peptide-bound class II or class I MHC complexes to elicit CD4 T helper or CD8 cytotoxic T lymphocyte responses, respectively. As stated before, PD-L1 is known to induce an exhaustion state in T cells, and to decrease the capability of a T cell-mediated response. The finding that several members of T cell activation pathway are downregulated in different T stages in OSCC indicates that T cell response is impaired either by the downregulation of important genes in this pathway, or even by the higher expression of PD-L1, causing an important reduction in T cell anti-tumor response. Therefore, rather than an indication of tumor immune-evasion, expression of PD-L1 by tumor cells might reflect the presence of ineffective anti-tumor immune pressure mediated by tumor infiltrating lymphocytes.

*HOXB9* is a direct transcriptional target of *WNT/TCF4*, and its expression is almost solely seen at embryonic stages. Nevertheless, HOXB9 expression was reported in pulmonary tumors, and its induction in pulmonary tumor cultured cells (H2030 e PC9) caused a rise in metastatic activity of these cell lines, resulting in a higher capacity of metastasizing to brain and bones [26, 27]. In breast tumors, Hayashida and co-workers showed that HOXB9 was capable of promoting neo-vascularization and distal metastasis in mice xenografts, suggesting that HOXB9 could contribute to breast cancer development due to induction of several growth factors, changing either the development of tumor cells and tumor stromal cells [28].

Although we have not found a relationship between HOXB9 expression and clinical and pathological parameters, the high number of HOX genes involved in OSCC development has drawn our attention. HOX family members have previously been implicated in OSCC. For example, HOXA1 was shown to be an independent prognostic factor in an OSCC cohort, and was also related with T stage, N stage, tumor differentiation and proliferative potential [29]. HOXB7 was shown to induce cellular proliferation in HaCAT human epithelial cell lines, and was also associated with overall survival. Regarding HOXB9 expression, to the best of our knowledge, this is the first report of HOXB9 expression in OSCC. HOXB9 transcript appears to important either in primary OSCCs and OSCC-derived CTCs, as we found the same tendency between the A.C. Camargo Cancer Center cohort and the Ohio State University cases.

Regarding CTCs, it is important to understand some problems with common separation methods (EPCAM-positive selection). First, it is important to remember that at least 33% of all patients with detectable metastatic disease do not appear to have any CTCs detectable by EPCAM-based methods [30]. Second, based on the high percentage of CTCs that may be unidentifiable by EPCAM-based selection methods, we could see a CTC as a circulating cell, with morphology compatible with a tumor cell, showing nucleus, negativity to pan-hematopoietic marker CD45 and positivity to CK8, CK18 and CK19, regardless of EPCAM status [31]. Thus, we sought to investigate the expression of the four markers identified by cDNA microarray in CTCs, using Nanostring, a multiplexed measurement of gene expression with color-coded probe pairs, with a sensitivity of less than one copy per cell, in CTCs from 4 patients enrolled at The Ohio State University Wexner medical Center, Columbus, OH [32]. Interestingly, the expression of PD-L1, HOXB9 and ZNF813 were similar to that found in primary tumors from A. C. Camargo Cancer Center, especially in larger tumors. We found their expression elevated in CTCs from OSCC patients, when compared to blood from healthy donors. For BLNK, we found a down-regulation in expression in CTCs, which parallels the decrease in its expression in primary tumors. We also investigated PD-L1 expression in CTCs from another 4 patients enrolled at the same institution and found a strong and focal staining pattern of PD-L1 immunofluorescence in CTCs, in a different pattern from what was seen in blood cells from healthy donors, as well, although the low number of samples did not allow us to make further consideration and analysis.

In this work, we believe we demonstrated an important role for PD-L1 expression in primary tumors according to tumor size, and in disease specific survival. Also, we demonstrate for the first time, the transcriptional and protein expression of PD-L1 in OSCC-derived CTCs, and transcriptional expression of HOXB9, ZNF813, and down-regulation of BLNK. Our findings shed light on the role of PD-L1 in OSCC development, especially considering that PD-L1 has received great attention due to its importance as a drug target in phase I and phase II trials. Although further work is needed to unveil the exact mechanism of PD-L1 in OSCC, we can speculate that the rise in PD-L1 expression in OSCC shows that at least some patients could benefit from anti-PD-L1 therapy in future. Also, we could further develop CTCs separation and characterization to identify individuals with PD-L1+ CTCs, and possibly monitor treatment response using CTCs.

## MATERIALS AND METHODS

### Ethics approval

The present study was approved by the Institutional Ethics Review Board of A. C. Camargo Cancer Center (approval number 1416/10), Ribeirao Preto Medical School (approval number 2401/2008), and Ohio State IRB following institutional and national guidelines. All samples used in this study were previously archived at the files of the Department of Pathology of A. C. Camargo Cancer Center, and from Department of Pathology of Ribeirao Preto Medical School. All written consents were obtained from all patients for the use of biological material, as well as for the use of their information, and storage at hospital database. When written consent was not possible to be obtained, according to national guidelines, the reasons for not doing so were provided to Institutional Ethics Review Board in order to obtain authorization for the use of samples.

### Case selection

For cDNA microarray and qRT-PCR analysis, 36 OSCC cases were selected randomly, based on T stage status from the Biobank of the Department of Anatomic Pathology from A.C. Camargo Cancer Center. All medical records were reviewed and analyzed to collect information regarding gender, tumor size at diagnosis, perineural invasion, angio-lymphatic invasion, and lymph node status of the patients.

For immunohistochemical analysis and validation of microarray data, a total of 142 cases of primary OSCCs diagnosed between 1990 and 2009 were retrieved from the medical files of the General Hospital of Ribeirao Preto Medical School, University of Sao Paulo, Brazil. The files were reviewed and analyzed to collect information regarding age, gender, smoking and alcohol intake history, primary tumor site, histological classification, treatment, tumor recurrences, regional and distant metastasis, disease-free survival (DFS) and overall survival (OS) of the patients.

The inclusion criteria for selection in validation group were as follows: 1) adequate clinicopathological data with sufficient follow-up; 2) the availability of sufficient tumor material; 3) the presence oral cavity and oropharyngeal cancer (including oral tongue, floor of the mouth, gingiva, buccal mucosa, hard palate, retromolar trigone and tonsil) (ICD-10: C00, C02-C06, C-09) no history of previous head and neck cancer; 5) no previous radio- or chemotherapy.

The patients were dichotomized by tobacco and alcohol consumption into “never consumer” and “current consumer” groups, according to previously described standardized criteria [31]. Similarly, tumor recurrence was defined as the occurrence of another carcinoma ≤ 2 cm away from the primary carcinoma [32], and the tumors were classified as either well-, moderately, or poorly differentiated according to the World Health Organization (WHO) histological differentiation grade classification [33]. For each case, all available hematoxylin and eosin (HE)-stained sections were reviewed to confirm the diagnosis of OSCC and to select a representative tumor area for Tissue Microarray (TMA) construction and immunostaining.

### RNA extraction

Approximately 30 mg of frozen tissue was homogenized with Precellys 24^®^ equipment (Carlsbad, California, USA). Afterward, the supernatant was used to purify total RNA with the RNeasy Mini kit (Qiagen, Venlo, the Netherlands) according to the manufacturer's protocol. The quantity and purity of RNA samples were assessed with NanoDrop™ ND-1000 (Thermo Scientific, Wilmington, Delaware, USA). RNA integrity was controlled with the Agilent Bioanalyzer 2100 (Agilent Technologies, Palo Alto, California, USA). Only RNA samples with OD 260/280 > 1.8 and RIN (RNA Integrity Number) > 6 were further processed.

### cDNA microarray experiment and analysis

The labeled cRNA from 36 samples was synthesized with the two-color microarray-based gene expression analysis Low Input Quick Amp-Labeling Kit (Agilent Technologies, Santa Clara, USA) following the manufacturer's standard protocol. Briefly, 250 ng of total RNA and the Agilent RNA Spike-in for two color (Agilent Technologies, Santa Clara, USA) were reverse-transcribed into double-stranded cDNA with the MMLV reverse transcriptase enzyme and primed with the oligo-dT-T7 polymerase promoter sequence. The Cy3-labeled cRNA obtained from cell culture pools, and Cy5-labeled cRNA obtained from OSCC samples were then transcribed *in vitro* by T7 RNA Polymerase. The quantity and efficiency of the labeled amplified Cy3-cRNA and Cy5-cRNA were determined with a NanoDrop™ ND-1000 (Thermo Scientific, Wilmington, Delaware, USA). Labeled cRNAs were hybridized to the Agilent G4851A SurePrint G3 Human GE 8x60k Microarray Kit (Agilent, Santa Clara, USA). Hybridizations were performed for 17 h at 10 rpm and 65°C in a hybridization oven (Agilent). After hybridization, the slides were washed and scanned at 5-μm resolution with the Agilent Bundle Microarray Scanner System (Agilent). Scanned image files were visually inspected for artifacts, and the fluorescence intensities were extracted and pre-processed with Agilent Feature Extraction software (v9.5.3.1).

Flagged spots were those not found, with low intensity, saturated, non-uniform (considering spot and background), obtained by low PMT (photomultiplier tube), considered outliers when compared with other spots of the same population (replicates) or background of these populations, and positive and negative controls. Spots included in the analysis were those flagged in a maximum of three samples of the all groups. Probes with replicates were averaged. All microarray raw data have been deposited in the GEO public database (http://www.ncbi.nlm.nih.gov/geo), a MIAME compliant database, under accession number GSE59069.

For microarray analysis, only the annotated genes were considered. Data were analyzed first by comparing T4 samples against T1 samples, in order to better determine which expression patterns were descending and ascending between these two groups, and then its behavior in T2 and T3 stages was further observed before model profiling.

### Model profiling

After filtering emission intensity data from each case in GeneSpring 12.6 software (Agilent Technologies), each case was classified according to its T stage. After hierarchical clustering, the means of gene expression for each group (T stage) were calculated, together with GeneID from each gene in the array. The differential expression between different tumor sizes was evaluated with STEM analysis (http://www.cs.cmu.edu/~jernst/stem/). The STEM clustering method initially defines a set of distinct and representative model of temporal expression profiles that correspond to possible profiles of a gene's expression change over time, independent of the data. All model profiles start at 0, and between two time points a model profile can hold steady, increase or decrease an integral number of units up to a parameter value. In our analysis, the parameter value was defined as any fold-change. Then, the log_2_ gene expression values of the time series experiment for each mRNA and lincRNA are normalized, so that T1 corresponds to 0 (T1 = 0), and T2, T3 and T4 stages are normalized accordingly. Each mRNA or lincRNA is assigned to the model profile to which its time series most closely matches based on the correlation coefficient. The number of mRNAs and/or lincRNAs assigned to each model profile is then computed. Also, the number of mRNAs and/or lincRNAs expected by chance to be assigned to a profile is calculated by randomly permuting the original time point values, renormalizing the expression values, then assigning mRNAs and/or lincRNAs to their most closely matching model profiles, and repeating for a large number of permutations. The average number of all permutations is used as the estimate of the expected number of mRNAs and/or lincRNAs assigned to the profile. The statistical significance of the number of mRNAs and/or lincRNAs assigned to each profile versus the number expected is then computed, indicating if the profile presents more or less mRNAs and/or lincRNAs than expected by chance.

### Ingenuity pathways analysis

Ingenuity Pathway Analysis software (IPA) (Ingenuity System Inc, USA) was used for interpretation of data in the context of biological processes, pathways and networks. Both up- and down-regulated genes were defined as value parameters for the analysis. After the analysis, generated networks are ordered by a score meaning significance. On the other side, significance of the biofunctions and the canonical pathways were tested by the Fisher Exact test.

For analysis of Canonical Pathways a list of genes taken from the dataset obtained after data filtering is noted in the different canonical pathways used by IPA database. The results were analyzed using Fisher's exact test to determine whether the number of genes included in the filtered data is greater than expected by chance in a given canonical pathway. The result of this analysis was evaluated by two measures: 1) ratio, that is the ratio between the number of genes of your dataset relative to the total number of genes in a given pathway; 2) *p*-value from Fisher's exact test.

### qRT-PCR and statistical methods

For qRT-PCR analysis, genes of interest were selected based on their significance in the cDNA microarray profiles, while also taking into consideration their importance in terms of new treatment options, or targets from drugs already in drug development pipeline, which could have a more direct impact and application of our results.

Total RNA was extracted from frozen tumor specimens as previously described (see RNA extraction section) and then reverse transcribed using High Capacity cDNA Reverse Transcription Kit with RNase Inhibitor (Invitrogen, Carlsbad, CA, USA) according to the manufacturer's instructions. mRNA expression in OSCC tissues were performed in BioRad CF96FX instrument. The probes used in this study were PD-L1 (TaqMan^®^ probe number: Hs01125301_m1), DHDH (TaqMan^®^ probe number Hs00205528_m1), HOXB9 (TaqMan^®^ probe number Hs00256886_m1), ZNF813 (TaqMan^®^ probe number Hs00975121_gH), BLNK (TaqMan^®^ probe number Hs00179459_m1).

These probes were evaluated in all of the patients included in this microarray analysis. 2 μg of total RNA was converted to cDNA according to the manufacturer's protocol. PCR was performed in a total reaction volume of 20 μl, including 10 μl Taqman Universal Master Mix (2x), 1 μl of TaqMan Probes, 2 μl of cDNA, 7 μl of double-distilled water. The quantitative real-time PCR reaction was set at an initial denaturation step of 10 min at 95 uC; and 95 uC (5 seconds), 60 uC (60 seconds in a total 40 cycles with a final extension step at 72 uC for 5 min. All experiments were done in duplicate, using as a control for RT-qPCR reactions a pool composed of 5 samples of normal oral epithelium. All samples were normalized to GAPDH. The median in each duplicate was used to calculate relative mRNAs concentrations (DCt =Ct median target gene - Ct median GAPDH). Expression fold changes were calculated using 2-DDCt methods [34], and for statistical purposes, we have considered a gene as up-regulated when its expression was two-fold higher than control and down-regulated when two-fold lower than control. Any value with less than a two-fold increase or decrease was considered as a normal expression. As a control for RT-qPCR reactions we have not used any cell lines, but rather a pool composed of 5 samples of normal oral epithelium.

### TMA construction

For each case, all available HE-stained sections were reviewed to confirm the diagnosis of OSCC and to delineate the most significant tumor region in each case for inclusion in the construction of a TMA paraffin block. Two tissue cylinders of each OSCC case (diameter, 1 mm) were punched from the selected regions of each of the 150 donor paraffin blocks and arrayed into a new recipient paraffin block using a Manual Tissue Arrayer I (Beecher Instruments, Silver Spring, USA). Three-micron-thick sections were cut from the TMA paraffin block using the Paraffin Tape-Transfer System (Instrumedics, Saint Louis, USA). One section was stained with HE to confirm the presence of the tumor, and the other sections were subjected to immunohistochemical (IHC) analysis.

### Immunohistochemistry

All of the tissue samples were fixed in 4% neutral formalin and embedded in paraffin. The IHC staining was performed using the Dako ENVISION system (Dako, Carpinteria, CA, USA). The sections were de-paraffinized in xylene and rehydrated through a series of graded alcohols. The endogenous peroxidase activity was blocked for 30 minutes in a solution containing 0.3% hydrogen peroxide. Antigen retrieval was performed by incubating the sections in a 10-mM citrate buffer for 40 minutes in a vapor lock. After antigen retrieval, the specimens were allowed to cool for 30 minutes and were then incubated at 4ºC overnight with the indicated primary antibodies: PD-L1 (1:25, goat, polyclonal, Abcam cat # ab28753, Cambridge, EUA) and HOXB9 (1:150, rabbit, polyclonal, Abcam cat # ab66765, Cambridge, USA). After an overnight incubation with the primary antibody, the slides were incubated with post-primary solution for 30 minutes and were then incubated with the polymer for 30 minutes (both provided by the Dako ENVISION system). The reactions were developed with diaminobenzidine (DAB), followed by Mayer's hematoxylin counterstaining. The slides were then dehydrated in a graded series of ethanol and mounted with Permount (Fischer, Fairlawn, NJ).

The slides were viewed under a microscope with image capture system (Leica DM4000B). For PD-L1, the cutoff chosen for membrane staining was 5% of tumor cells [35]. For cytoplasmic staining, we chose to use the same cut-off due to lack of studies using PD-L1 expression in cytoplasm. To HOXB9, we use a variation of the model proposed by Sha in gastric carcinomas [36]. The cases were dichotomized using a cut-off of 50% of tumor cells with nuclear staining.

### Statistical analysis

The statistical analyses and tests were performed with the commercially available IBM SPSS Statistics 21.0 software (Chicago, IL). Descriptive statistics were used to summarize the study data. The DFS period was defined as the time from diagnosis until the occurrence of local recurrence or metastasis (the metastases were confirmed through histopathological analysis), and OS period was defined as the interval between the diagnosis and the date of death for the uncensored observations or the date of the last information recorded in the medical records for censored observations. To evaluate the prognostic significance of PD-L1 and HOXB9 protein expression in OSCCs, the data was analyzed together with the clinicopathological features through cross tables using Fisher's exact or chi-square tests. A multivariate Cox proportional hazard regression model was used to build models containing a subset of candidate risk factors with independent prognostic properties, and as well as the Kaplan-Meier method and the log-rank test to estimate and compare the cumulative survival rates after multivariate analysis. Statistical significance was defined as a two-tailed *P*-value ≤ 0.05.

### Cell cultures

Breast cancer cell lines, MDA-MB 231 (HTB-26), and tongue cancer cell line, SCC-4(CRL-1624), were procured from ATCC (Manassas, VA). These cells were grown to mid-log phase in Dulbecco's Modified Eagle Medium (DMEM) (Cellgro) with 10% fetal bovine serum (FBS) (Invitrogen) and 1% nonessential amino acid (Cellgro) at 37°C in 5% CO2 atmosphere. Cell lines were harvested by washing the adherent cells with phosphate buffer saline (PBS) and subsequently incubating with Accutase (Innovative Cell Technologies, Inc.) for 10 min at 37°C to detach cells from the culture-flasks. Accutase was then neutralized with the culture medium before pelleting the cells at 350xg for 5 min. Cells were resuspended in culture medium for downstream experiments.

### Patient samples and blood collection

Patients with diagnosis of oral and oropharynx squamous cell carcinoma, older than 18 years of age, were enrolled in Institutional Review Board (IRB) approved protocols that permit CTC characterization of blood specimens. All patients gave their informed consent to participate in the study. Blood samples were collected, after several standard blood tubes were drawn for routine chemotherapy labs, either prior to initiation of a new line of systemic therapy or at progression. Peripheral blood (7.8 to 17.7 mL) was collected in BD Vacutainer tubes (BD Biosciences) for CTC enumeration and processed within 4 hours of blood collection.

### Normal control blood collection

Healthy donor blood was collected from volunteer donors after obtaining informed consent using an IRB-approved protocol at The Ohio State University Medical Center and processed in the same manner as the patient samples. Source leukocytes (Buffy coat) were purchased from American Red Cross, Central-Southeast Ohio region.

### Sample processing for negative depletion enrichment

Blood samples were kept at ambient temperature and processed within 4 hours after blood drawing using an immunomagnetic negative depletion methodology, as described previously [37]. Briefly, blood samples were subjected to a red blood cell lysis step and labeling with anti-CD45 tetrameric antibody complex (TAC; Stem Cell Technologies). Magnetic nanoparticles were added and incubated with the cell suspension that subsequently runs through the quadrupole magnetic sorter system (QMS). The effectiveness of the negative magnetic enrichment (NME) on patient blood was evaluated as the log_10_ of the ratio of the total nucleated cell number before QMS to that after QMS.

Nucleated cell counts were determined prior to RBC lysis step, after RBC lysis and after NME of CD45+ depleted labeled cells. Lysis efficiency, nucleated cell log depletion and total cell log depletion were used to evaluate the performance of the NME methodology. The algorithm for these three parameters was introduced in our earlier publication [37]. As previously reported, more than 120 blood specimens from breast cancer patient have been processed with this methodology.

### Nanostring analysis

Approximately 20,000 cells from QMS were lysed in 5 μL of lysis buffer RLT (RNeasy Kit, Qiagen) and stored in aliquots at −80°C. The system used to determine the expression profile of target genes in CTCs was NanoString nCounter (Nanostring Technologies). Briefly, a multiplexed probe library is made with two sequence-specific probes for each gene of interest. The first probe, a capture probe, containing a 35- to 50-base sequence complementary to a particular target mRNA plus a short common sequence coupled to an affinity tag such as biotin. The second probe, the reporter probe, contains a second 35- to 50-base sequence complementary to the target mRNA, which is coupled to a color-coded tag that provides the detection signal. The tag consists of a single-stranded DNA molecule, the backbone, annealed to a series of complementary *in vitro* transcribed RNA segments each labeled with a specific fluorophore. The linear order of these differently colored RNA segments creates a unique code for each gene of interest. The cartridge containing the probes used in this study consisted of 44 probes representing 41 genes and three controls. Each sample was hybridized in duplicate or triplicate using approximately 20,000 cells obtained after magnetic separation process with the QMS.

### CTC characterization and immunofluorescence

Previously processed cells (described above) were centrifuged to a glass slide using Cytospin (Thermo Scientific) and were hydrated in PBS for 5 min at room temperature. After washing with PBS, cells were incubated with monoclonal antibody anti-CD45 raised (1:50, mice, Clone HI30, BD Pharmingen, cat # 555480) with polyclonal antibody anti-PD-L1 raised in rabbit (5 ug/ml, rabbit, polyclonal, Abcam, cat # ab58810) in antibody dilution solution to reduce background labeling (Dako, Carpinteria, USA, cat # S3022) for 1 hour at room temperature. After staining with primary antibody, the slides were washed in PBS for three cycles of 5-minute each. Secondary antibodies Alexa Fluor^®^ 647, anti-mouse IgG (H + L) (1: 400, Invitrogen, cat # A-31571) and Alexa Fluor^®^ 594 anti-rabbit IgG (H + L) (1: 200 Invitrogen, cat # A-21207), were added for 1 hour at room temperature. Subsequently, cells were permeabilized with 0.25% Triton X-100 (T-100) in PBS for 15 min at room temperature and then blocked with 1% BSA in PBS for 1 hour at room temperature and washed PBS. Customized anti-cytokeratin antibody (Myltenyi Biotec, CK3–6H5 clone) conjugated to Alexa Fluor^®^ 488 anti-mouse IgG (H + L) (1: 200, goat, Invitrogen, cat # A11029) for 1 hour at room temperature. After further washing with PBS, the slide was mounted using the ProLong^®^ Gold antifade mounting medium with DAPI (Invitrogen, cat # P36941) and observed with a Nikon I90 (Nikon) fluorescence microscope. The cell line MDA-MB-231 was previously used to develop the staining protocols and act as a positive control for all immunofluorescence tests used in this study.

## SUPPLEMENTARY TABLES






