# Supplementary material for: Gene expression patterns through oral squamous cell carcinoma development: PD-L1 expression in primary tumor and circulating tumor cells
Source: Oncotarget. 2015 May 15;6(25):20902–20. doi: 10.18632/oncotarget.3939 (PMC4673238; doi:10.18632/oncotarget.3939)
Supplement: Supplementary file 1 [file oncotarget-06-20902-s001.pdf]

SUPPLEMENTARY TABLES

Supplementary Table 1: Networks identified by IPA, with information of molecules present in dataset and included in the networks

Supplementary Table 2: Ingenuity Canonical Pathways, including pathways, -log (*p*-value), *p*-value, ratio, z-score and molecules
